# Primary Bone Diffuse Large B-cell Lymphoma (DLBCL) in a Young Adult: A Case Report

**DOI:** 10.7759/cureus.110991

**Published:** 2026-06-16

**Authors:** Manal Derkaoui, Anass Haloui, Hajar Mbarki, Nassira Karich, Amal Bennani

**Affiliations:** 1 Pathology, Faculty of Medicine and Pharmacy of Oujda, Mohammed First University of Oujda, Oujda, MAR; 2 Internal Medicine, Faculty of Medicine and Pharmacy of Oujda, Mohammed First University of Oujda, Oujda, MAR

**Keywords:** diffuse large b-cell lymphoma, dlbcl, immunohistochemistry, primary bone lymphoma, r-chop, young adult

## Abstract

Primary bone diffuse large B-cell lymphoma (DLBCL) is a rare subtype of extranodal non-Hodgkin lymphoma that predominantly involves long bones, particularly the metaphyseal regions. We report the case of a 23-year-old man presenting with painful swelling of the right knee. Imaging revealed an osteolytic lesion of the distal right femur with cortical destruction and soft tissue extension. Staging contrast-enhanced computed tomography of the chest, abdomen, and pelvis showed no nodal or visceral involvement, consistent with localized (limited-stage) disease. Histopathological examination of the biopsy specimen demonstrated diffuse sheets of large atypical lymphoid cells, and immunohistochemical analysis confirmed DLBCL with a non-germinal center B-cell phenotype. The patient received six cycles of R-CHOP chemotherapy (rituximab, cyclophosphamide, doxorubicin, vincristine, and prednisone), resulting in rapid clinical improvement and complete metabolic remission on post-treatment positron emission tomography, consistent with a complete response. This case underscores the importance of including lymphoma in the differential diagnosis of destructive bone lesions in young adults and highlights the favorable prognosis of localized primary bone DLBCL with appropriate chemoimmunotherapy.

## Introduction

Primary bone lymphoma (PBL) is defined as a malignant lymphoid neoplasm presenting with one or more bone lesions without evidence of nodal or other extranodal disease at diagnosis, according to the World Health Organization (WHO) classification [1]. PBL is, by definition, confined to the skeleton at presentation, which distinguishes it from secondary skeletal involvement by a disseminated systemic lymphoma; staging according to the Lugano classification is used to confirm the absence of nodal or other extraosseous disease. Most PBLs correspond to non-Hodgkin lymphomas, of which diffuse large B-cell lymphoma, not otherwise specified (DLBCL, NOS), represents the predominant histologic subtype, accounting for approximately 80% of cases [2]. The femur is the most frequently involved site, accounting for approximately 29% of cases, followed by the pelvis, humerus, skull, and tibia [3].

PBL is a rare entity, representing less than 1% of all lymphomas and approximately 3-15% of extranodal lymphomas [4]. It predominantly affects middle-aged adults between the fifth and sixth decades of life, and its occurrence in young adults is unusual, potentially leading to diagnostic delay. The nonspecific clinical and radiological presentation, often mimicking primary bone sarcomas or inflammatory conditions, underscores the essential role of histopathology and immunohistochemistry in establishing the diagnosis.

Recognising this entity is clinically important because, unlike the bone sarcomas and infections it frequently mimics, primary bone DLBCL is highly responsive to chemoimmunotherapy and carries a comparatively favourable prognosis, so that an accurate and timely diagnosis directly changes management. The aim of this report is to describe a case of primary bone DLBCL in a 23-year-old man, emphasizing its clinical, radiological, and pathological features, and to review the current literature on this rare entity.

## Case presentation

A 23-year-old man with no significant past medical history presented with painful swelling of the right knee associated with inflammatory pain. Physical examination revealed a warm, erythematous mass with limited and painful joint mobility. The patellar tap test was positive. No peripheral lymphadenopathy was detected.

Plain radiography demonstrated an osteolytic lesion of the distal femur with adjacent soft tissue thickening. Computed tomography (CT) scan showed an epiphyseal-metaphyseal-diaphyseal lesion of the distal right femur with focal cortical destruction and extension into adjacent soft tissues, particularly the prefemoral fat, displacing the vastus intermedius and medialis muscles (Figure 1). Contrast-enhanced CT of the chest, abdomen, and pelvis revealed no nodal or visceral involvement. Given the patient's young age and the aggressive osteolytic appearance of the lesion, the clinical and radiological differential diagnosis included osteosarcoma, Ewing sarcoma, osteomyelitis, and Langerhans cell histiocytosis, which were subsequently excluded on histopathological and immunohistochemical examination.

**Figure 1:**
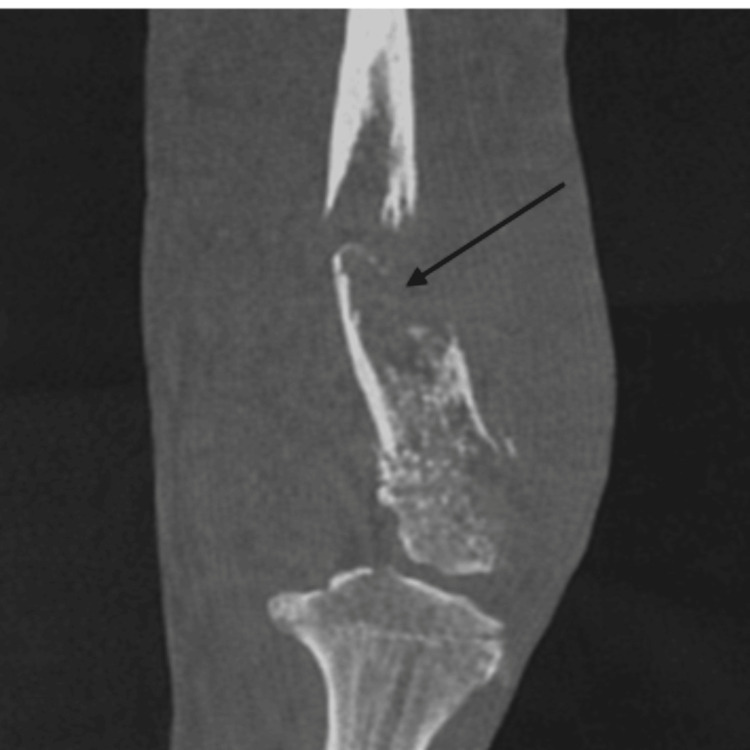
Sagittal CT reconstruction of the distal femur showing an osteolytic lesion with poorly defined borders involving the distal metaphysis, associated with a cortical fracture and extension into the adjacent soft tissues (arrow).

A biopsy of the mass was subsequently performed. Histological examination showed diffuse sheets of large atypical lymphoid cells. The tumor cells displayed irregular nuclei with vesicular chromatin and occasional prominent nucleoli. The cytoplasm ranged from clear to eosinophilic. Numerous mitotic figures were identified. The stroma was fibro-inflammatory with scattered tingible-body macrophages (Figure 2).

**Figure 2:**
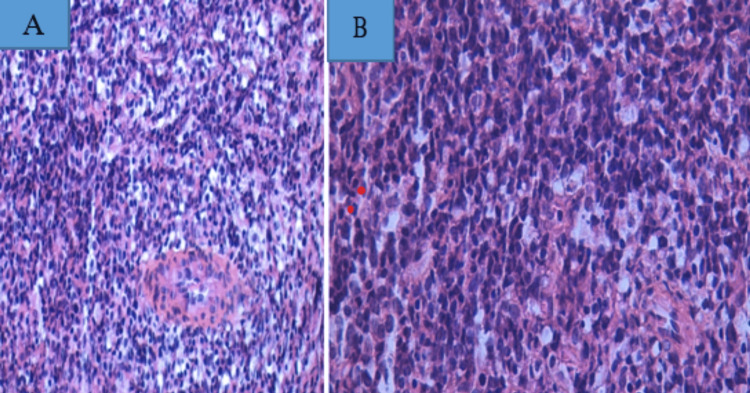
Histopathological examination showing a diffuse proliferation of large atypical discohesive cells arranged in sheets. (A) H&E stain, ×20 magnification. (B) H&E stain, ×40 magnification.

Immunohistochemistry demonstrated strong diffuse expression of B-cell markers CD20, CD79a, and PAX5, confirming B-cell lineage, along with BCL2 positivity. Tumor cells expressed BCL6 and MUM1, while CD10 was negative, consistent with a non-germinal center B-cell (non-GCB) phenotype according to the Hans algorithm. BCL6 and BCL2 expression showed heterogeneous staining among the neoplastic cells. Cyclin D1, CD23, and CD138 were negative, excluding mantle cell lymphoma, chronic lymphocytic leukemia/small lymphocytic lymphoma, and plasmacytic differentiation, respectively. CD99 also showed heterogeneous expression. Myogenic and myeloid markers, including myogenin and myeloperoxidase (MPO), were negative. The Ki-67 proliferation index was approximately 90%, indicating a highly proliferative tumor. Background reactive CD5-positive T lymphocytes were present. Overall, these findings support the diagnosis of DLBCL, NOS.

**Figure 3:**
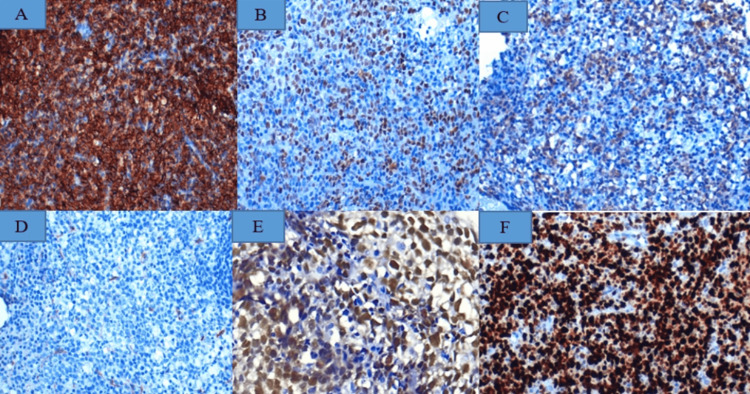
Immunohistochemical staining of diffuse large B-cell lymphoma, not otherwise specified (DLBCL, NOS) (×40). Tumor cells show strong positivity for (A) CD20, confirming B-cell lineage. (B) BCL6 and (C) BCL2 expression are heterogeneous among the neoplastic cells. (D) CD10 is negative. (E) MUM1 shows positive nuclear staining in the tumor cells, supporting an activated B-cell/post-germinal center phenotype. The Ki-67 proliferation index (F) is markedly elevated, estimated at approximately 90%, reflecting high proliferative activity.

Based on histopathological and immunophenotypic findings, a diagnosis of primary bone DLBCL was established. Staging CT showed no nodal or extranodal disease. The patient received six cycles of R-CHOP chemotherapy (rituximab, cyclophosphamide, doxorubicin, vincristine, and prednisone), with marked clinical improvement after the fourth cycle, including positron emission tomography (PET) scan demonstrated complete metabolic remission.

A summary of findings in this case is shown in Table 1.

**Table 1:** Summary of the clinical, radiological, histopathological, immunophenotypic, and treatment findings in the present case. IHC: immunohistochemistry, NOS: not otherwise specified, R-CHOP: rituximab, cyclophosphamide, doxorubicin, vincristine, and prednisone, MPO: myeloperoxidase

Parameter	Finding
Age / sex	23-year-old man
Presentation	Painful swelling of the right knee with inflammatory pain; warm, erythematous mass; positive patellar tap; no peripheral lymphadenopathy
Site	Distal right femur (epiphyseal-metaphyseal-diaphyseal)
Radiograph / CT	Osteolytic lesion with focal cortical destruction and soft-tissue extension
Staging CT (chest/abdomen/pelvis)	No nodal or visceral involvement; localized (limited-stage) disease
Histology	Diffuse sheets of large atypical lymphoid cells with a high mitotic rate
IHC - positive	CD20, CD79a, PAX5, BCL6, BCL2, MUM1
IHC - negative	CD10, cyclin D1, CD23, CD138, myogenin, MPO
Ki-67 index	Approximately 90%
Cell of origin (Hans)	Non-germinal-center B-cell phenotype by CD10/BCL6, MUM1
Diagnosis	Primary bone diffuse large B-cell lymphoma, NOS
Treatment	Six cycles of R-CHOP
Outcome	Complete metabolic remission on PET

## Discussion

Primary bone DLBCL represents a rare clinicopathological phenomenon that represents 1% or fewer of lymphomas and about 3% to 15% of extranodal lymphomas [4]. Primary bone DLBCL is characterized as a lymphoma occurring within the bone, with no signs of systemic disease and distant nodal involvement during its presentation [5]. Staging according to the Lugano classification is recommended to confirm the absence of nodal and systemic involvement. The differentiation is of great importance due to different biological characteristics, treatment strategies, and prognosis compared with disseminated secondary bone lymphoma from systemic lymphoma.

PBL usually presents itself in middle-aged patients, especially those aged between the fifth and sixth decade, with slight male predilection. In our report, PBL occurrence in younger individuals is an unusual finding and can contribute to delayed diagnosis, since PBL is not among the first diagnoses considered for younger patients who present with bone lesions. Pain is the most common initial complaint, which accounts for over 80% of cases, while swelling and localized tenderness occur in 20% to 30%. In contrast to nodal lymphomas, constitutional "B" symptoms (fever, night sweats, and weight loss) rarely occur. PBL can have a structural impact on any bone in the body, but it is mostly found in the long bones like the femur, humerus, and tibia [6]. It is suggested that metaphyseal involvement is related to the number of blood vessels available there, along with the role of medulla as a hematopoietic environment [7].

Imaging findings in PBL are nonspecific and variable. On conventional radiographs and CT, the disease most often produces a permeative or moth-eaten osteolytic pattern arising from the medullary cavity, which may be accompanied by cortical destruction and an associated soft-tissue mass; magnetic resonance imaging typically reveals marrow infiltration that is frequently more extensive than the abnormalities apparent on radiographs. In terms of relative frequency, osteolytic lesions predominate (approximately 77% of cases), whereas purely sclerotic (about 4%) and mixed lytic-sclerotic (about 16%) patterns are uncommon. Because these appearances overlap substantially with those of other primary bone malignancies, imaging alone is insufficient for diagnosis and histopathological confirmation is required [8].

The most common type of lymphoma involved in PBL is non-Hodgkin lymphoma, which makes up more than 90% of cases, among which the most common subtype is DLBCL, NOS, comprising almost 80% of PBLs. Other subtypes of PBL are follicular lymphoma, marginal zone lymphoma, small lymphocytic lymphoma (SLL), lymphoplasmacytic lymphoma, Burkitt lymphoma, B-lymphoblastic lymphoma, anaplastic large cell lymphoma (ALCL), adult T-cell lymphoma/leukemia (ATLL), extranodal NK/T-cell lymphoma, and peripheral T-cell lymphoma (PTCL), NOS [7].

Histologically, primary bone DLBCL presents as diffuse sheets of large abnormal lymphoid cells with prominent vesicular nuclei, prominent nucleoli, and brisk mitotic rate. Histological confirmation of the disease is achieved through the use of immunohistochemical staining, which is crucial for determination of cell type and exclusion of mimickers. Primary bone DLBCL expresses B-cell-associated markers including CD20, CD79a, and PAX5, whereas co-expression of CD10, BCL6, and MUM1 helps classify the tumor into GCB or non-GCB phenotype based on immunohistochemistry scoring algorithms such as the Hans criteria. Although a non-GCB phenotype is associated with less favourable outcomes in systemic DLBCL, the prognostic relevance of cell-of-origin classification in primary bone DLBCL remains uncertain owing to the small number of reported cases, and the prognostic value of the Hans algorithm itself has been questioned in the rituximab era; these findings should therefore be interpreted with caution [1].

Genetic alterations are also vital for understanding the biology of malignant lymphomas. Abnormalities involving rearrangements of BCL2, MYC, or BCL6 genes are common in DLBCL patients and contribute to the prediction of disease behavior and therapy choice. Lymphomas with the expression of both MYC and BCL2 antigens are referred to as double-expressor lymphomas and are considered more clinically aggressive in systemic disease. Unfortunately, the genetic spectrum of primary bone DLBCLs is understudied due to the paucity of cases with this condition [9]. Consequently, the prognostic and biological significance of these alterations in primary bone DLBCL remain largely extrapolated from systemic disease and await confirmation in larger series.

In therapeutics, modern treatment approaches mimic that for nodal DLBCL. Anthracycline-containing chemoimmunotherapies (R-CHOP) remain the backbone of modern treatment and have considerably improved survival rates. In particular, a number of retrospective trials have shown the superiority of progression-free survival rate in patients receiving regimens involving rituximab in comparison to chemotherapy alone [6]. The contribution of post-chemotherapy radiotherapy to survival is still controversial; some series report better results, but other reports failed to prove its value. Treatment decision is usually tailored depending on the clinical situation [1].

Surgery is mainly restricted to diagnostic procedures and orthopedic stabilization in case of pathological fracture or increased risk of fractures. Since bone structural integrity may be compromised despite tumor regression, orthopedic consultation should be performed throughout treatment and recovery process [3].

As concerns the prognosis, primary bone DLBCL can be regarded as one of the extranodal subtypes with the best outlook. Five-year overall survival in localized tumors approaches 90%. Factors affecting prognosis include well-known indices of lymphoma prognosis, such as stage of disease, performance score, serum lactate dehydrogenase level, and international prognostic index (IPI) [10].

The current case emphasizes the need to consider the diagnosis of lymphoma as one of the possible causes of osteolysis, particularly in young patients. Moreover, histopathologic and immunohistopathologic examination plays a critical role in making an accurate diagnosis since plain radiographic studies fail to identify the underlying condition. Timely treatment with chemoimmunotherapy can result in favorable clinical outcomes.

This report has several limitations inherent to a single case. Molecular studies, including fluorescence in situ hybridisation (FISH) for MYC, BCL2, and BCL6 rearrangements, were not performed; therefore, high-grade B-cell lymphoma with double-hit or triple-hit genetic abnormalities cannot be excluded. Further morphological subclassification (e.g., centroblastic versus immunoblastic) was not performed. Baseline laboratory data, including serum lactate dehydrogenase levels, and the exact duration of symptoms prior to diagnosis were not available. These limitations should be taken into account when interpreting the findings.

## Conclusions

Primary bone DLBCL in young adults is an uncommon presentation that may represent a diagnostic challenge because of its nonspecific clinical and radiologic features, often mimicking primary bone tumors or inflammatory conditions. Definitive diagnosis relies on histopathological and immunohistochemical evaluation to confirm the lymphoid origin and subtype. Early recognition is essential, as this entity is highly responsive to combined chemoimmunotherapy and generally carries a more favorable prognosis than most primary bone malignancies. In the present case, several favourable prognostic factors were present, including young age, good performance status, localized (limited-stage) disease, and a complete metabolic response to R-CHOP. This case emphasizes the importance of including lymphoma in the differential diagnosis of destructive bone lesions in young patients and highlights the value of a multidisciplinary approach for optimal management.
